# Zinc solubilizing bacteria and their potential as bioinoculant for growth promotion of green soybean (*Glycine max* L. Merr.)

**DOI:** 10.7717/peerj.15128

**Published:** 2023-05-10

**Authors:** Moltira Srithaworn, Jieb Jaroenthanyakorn, Janpen Tangjitjaroenkun, Chanwit Suriyachadkun, Orawan Chunhachart

**Affiliations:** 1 Division of Microbiology, Department of Science and Bioinnovation, Faculty of Liberal Arts and Science, Kasetsart University Kamphaeng Saen Campus, Nakhon Pathom, Thailand; 2 Department of Resources and Environment, Faculty of Science at Sriracha, Kasetsart University Sriracha Campus, Chonburi, Thailand; 3 Thailand Bioresource Research Center, National Center for Genetic Engineering and Biotechnology (BIOTEC), National Science and Technology Development Agency (NSTDA), Patumthani, Thailand

**Keywords:** Zinc-solubilizing bacteria, *Priestia megaterium*, *Priestia aryabhattai*, *Bacillus*, Green soybean

## Abstract

Zinc-solubilizing rhizobacteria can convert insoluble zinc to an accessible form and increase Zn bioavailability in soil, which help mitigate Zn deficiency in crops. In this work, 121 bacterial isolates were isolated from the rhizosphere soils of peanuts, sweet potatoes, and cassava, and their capability to solubilize Zn was evaluated using Bunt and Rovira’s agar containing 0.1% ZnO and ZnCO_3_. Among these isolates, six showed high Zn solubilization efficiencies ranging from 1.32 to 2.84 and 1.93 to 2.27 on the medium supplemented with 0.1% ZnO and ZnCO_3_, respectively. In a quantitative analysis of soluble Zn in liquid medium supplemented with 0.1% ZnO, the isolate KAH109 showed the maximum soluble zinc concentration of 62.89 mg L^−1^. Among the six isolates, the isolate KAH109 also produced the most indole-3-acetic acid (IAA) at 33.44 mg L^−1^, whereas the isolate KEX505 also produced IAA at 17.24 mg L^−1^ along with showing zinc and potassium solubilization activity. These strains were identified as *Priestia megaterium* KAH109 and *Priestia aryabhattai* KEX505 based on 16S rDNA sequence analysis. In a greenhouse experiment conducted in Nakhon Pathom, Thailand the ability of *P. megaterium* KAH109 and *P. aryabhattai* KEX505 to stimulate the growth and production of green soybeans was examined. The results revealed that inoculation with *P. megaterium* KAH109 and *P. aryabhattai* KEX505 considerably increased plant dry weight by 26.96% and 8.79%, respectively, and the number of grains per plant by 48.97% and 35.29% when compared to those of the uninoculated control. According to these results, both strains can be considered as a potential zinc solubilizing bioinoculant to promote the growth and production yield of green soybeans.

## Introduction

Thailand’s important economic and food crops are tuber and legume plants, such as cassava, soybeans, and peanuts. According to the Office of Agricultural Economics (OAE), Thailand produced approximately 35 million tons of cassava, 50 thousand tons of soybeans, and 35 thousand tons of peanuts in 2021 (OAE, 2021). Zinc insufficiency is a common issue in Thailand, where calcareous soils are widely utilized for cultivating staple crops (Takrattanasaran et al., 2010; Chittamart et al., 2016). These soils have less bioavailable zinc because some of the zinc is adsorbed on the calcium carbonate in the calcareous soils (Takrattanasaran et al., 2013). In addition, Zn deficiency in crops is caused by a combination of factors, including low Zn levels in the soil, inadequate Zn solubility in the soil, and Zn conversion to an insoluble form (Sharma et al., 2013).

Zinc is one of the most important micronutrients required for plant growth, development, disease defense and resistance against stress (Cabot et al., 2019; Hassan et al., 2020). Zn is an essential component of many biomolecules, including lipids, proteins, and cofactors of auxins. Zn plays an important role in the metabolism of carbohydrates, synthesis of proteins and chlorophyll, protection of membrane lipids from reactive oxygen species, and biosynthesis of plant growth hormones such as auxin (Tsonev & Lidon, 2012). Zinc deficiency in plants results in smaller amounts of indole-3-acetic acid (IAA) in the shoot apical meristems and young leaves (Cakmak, Marschner & Bangerth, 1989). Young leaves with interveinal chlorosis, older leaves with spots, smaller leaves, and stunted development are signs of zinc deficiency in plants (Marschner, 2002). Zinc sulfate (ZnSO_4_) has been utilized extensively as an inorganic fertilizer for soil application due to its high solubility and low cost (Hussain, Maqsood & Rahmatullah, 2010). However, soluble zinc is rapidly transformed into various inaccessible forms in soils with generally poor characteristics, such as high pH values, calcium carbonate contents, or phosphate contents; therefore, the absorption of available zinc provided by zinc sulfate by plants shows low efficiency (Zhao et al., 2015). In addition to applying Zn fertilizer to the soil, zinc is also applied directly to crop leaves as a complementary approach for increasing the Zn content of crops (Doolette et al., 2018).

Zn-solubilizing rhizobacteria are a useful alternative to enhance zinc availability in the soil (Upadhayay et al., 2022; Saravanan, Kumar & Sa, 2011). Several zinc-solubilizing bacteria, such as *Pseudomonas protegens* RY2 (Yasmin et al., 2021), *Bacillus megaterium* (Bhatt & Maheshwari, 2020) and *Bacillus altitudinis* (Kushwaha et al., 2021), have been recognized as plant growth-promoting bacteria due to the production of plant hormones and growth factors and making zinc available to plants, which is beneficial for plant growth (Garcia & Kniffin, 2018). Furthermore, Zn-regulated transporters and iron (Fe)-regulated transporter-like protein (ZIP) genes are up regulated by *Enterobacter cloacae* ZSB14, a zinc-solubilizing strain. These genes are important for the transport and accumulation of Zn in rice in iron-deficient conditions (Krithika & Balachandar, 2016). Strains of zinc-solubilizing *Bacillus* spp. can be inoculated into the soil alone or in combination with chemical fertilizers to increase the soil zinc availability for soybean (Sharma et al., 2012) and wheat (Ali et al., 2021; Ramesh et al., 2014). It has also been suggested that seed priming or coating with zinc-soluble *Bacillus* is an alternative method for promoting plant development (Bhatt & Maheshwari, 2020; Kushwaha et al., 2021).

Green soybeans or edamame in Japanese (*Glycine max* L. Merr.) are some of the most significant grain legumes (Keatinge et al., 2011). In recent decades, the production of green soybeans has expanded considerably owing to the growing recognition of their nutritional benefits and a shift in lifestyles toward the consumption of healthier foods. However, stunted development, interveinal chlorosis of the leaves, and other signs of zinc shortage are present in green soybean cultivated on sandy soils, calcareous soils, and soils with high levels of phosphate (Hellal & Abdelhamid, 2013). Inorganic zinc fertilizers, such as ZnSO_4_ have been used to alleviate this deficit (Joy et al., 2015). However, the increased usage of chemical fertilizers has had negative impacts on the environment and people’s health (Guo et al., 2021). By increasing the amount of available zinc in the rhizosphere, zinc-solubilizing bacteria have the potential to be employed as bioinoculants or biostimulants to enhance plant growth and productivity. Therefore, the objective of this study was to isolate ZSB from the rhizosphere soils of peanut, sweet potato, and cassava. In addition, this study aimed to investigate ZSB at the molecular level and determine how they affect the growth of a greenhouse-grown green soybean plant model (*Glycine max* L. Merr).

## Materials & Methods

### Isolation of zinc-solubilizing bacterial strains

Rhizosphere soil samples were obtained from peanut (*Arachis hypogaea* L.), sweet potato (*Ipomoea batatas* L.) and cassava (*Manihot esculenta* L.) fields in Kanchanaburi, Chonburi and Rayong Provinces, Thailand, for the isolation and screening of Zn-solubilizing bacteria. The samples were collected from December 2020 to February 2021. Soil around the rhizosphere of plants at a depth of 10 to 15 cm was collected in sterile, dry polythene bags and transported to the laboratory for further analysis within 6 to 8 h after collection. The soil samples were ground into a powder after being air-dried. The soil sample containers were then filled with sterile water and agitated for two hours at 150 rpm. After that, the samples were allowed to settle for 5 min before being serially diluted up to 10^6^ times in one mL of suspension. Each dilution was applied to a nutrient agar plate and incubated at 30 ± 2 °C. The growing colonies were inspected, and bacterial colonies with various morphologies, such as white and creamy, yellowish white, smooth, glistening, rhizoid and opaque colonies, were chosen and purified using nutrient agar streaking.

### Zn solubilization assay

The ability to solubilize zinc was evaluated on Bunt and Rovira’s agar, and the pH was maintained at 7.2. Different sources of insoluble zinc salts, such as zinc oxide (ZnO) and zinc carbonate (ZnCO_3_) were supplemented individually at a final concentration of 0.1% to the medium, and the resulting medium was sterilized at 121 °C for 20 min. All tested strains were point inoculated onto the medium, and the plates were incubated at 30 ± 2 °C for 10 days. Strains with a distinct zone around the colony were termed zinc-solubilizing. The halo zone was measured, and the zinc solubilization efficiency (ZSE) of the strains was calculated as described by Yasmin et al. (2021) using the following equation:

ZSE = (HZ/C) ×100, where ZSE is the Zn solubilization efficiency, HZ is the diameter of the solubilization halo zone, and C is the diameter of the colony.

### Determination of soluble Zn

The quantitative measurement of soluble zinc was performed in a 150 mL conical flask containing 50 mL of Bunt and Rovira’s medium and 0.1% ZnO, which was incubated at 30 ± 2 °C with 150 rpm shaking. Ten percent log-phase inoculum was used. Uninoculated medium was used as a control. After 10 days of cultivation, cells were removed by centrifugation at 10,000 rpm for 10 min. The supernatant was collected and digested with HNO_3_ and HClO_4_ (1:4 v/v). To measure the quantity of soluble zinc, the digested sample was analyzed using an atomic absorption spectrophotometer (Agilent Technologies 200 Series AA, USA).

### Identification of selected strains

Morphological characteristics and Gram staining were carried out for identification. Then, the selected bacterial strains were identified through 16S rRNA gene sequencing. Genomic DNA (gDNA) was isolated from bacterial isolates using the GF-Bacterial DNA Extraction kit (Vivantis, Selangor, Malaysia) according to the manufacturer’s instructions. The gDNA was visualized using agarose gel electrophoresis, and the concentration of gDNA was determined using a NanoDrop DS-11 Series Spectrophotometer (DeVonix Inc., Wilmington, DE, USA). The 16S rRNA gene was amplified using the extracted gDNA. The primers fD1 (5′AGAGTTTGATCCTGGCTCAG 3′) and rP2 (5′ACGGCTACCTTGTTACGACTT 3′) were used for amplification of the 16S rRNA gene using polymerase chain reaction (PCR) (Weisburg et al., 1991). The PCR products were run on an agarose gel containing 1.5% agarose, purified using the GF-1 PCR Clean-up kit (Vivantis, Selangor, Malaysia), and sequenced commercially (1st BASE, Singapore). The 16S rRNA gene sequences of bacterial isolates were analyzed at the NCBI (the US National Center for Biotechnology Information) using BLAST against 16S ribosomal RNA sequences to determine the closest homologs. The 16S rRNA sequences with homologs showing 100% or 99% shared identity were chosen. The sequences of closely related type strains from the GenBank database were multiple-aligned using ClustalW in BioEdit Sequence Alignment Editor 7.2.5 for phylogenetic analysis (Hall, 1999). After removing gaps and ambiguous nucleotides from the sequences and performing multiple alignments, a phylogenetic tree was created using the maximum likelihood (ML) procedure in MEGA version 11 (Kumar et al., 2018) based on a comparison of 1,381–1,439 nucleotides present in all the strains. The sequence of *Escherichia coli* ATCC 11775^T^ (X80725) was used as an outgroup. Branches of the phylogenetic tree were assigned confidence levels using bootstrap analyses based on 1,000 resamples (Felsenstein, 1985).

### *In vitro* screening for growth promotion properties of Zn-solubilizing isolates

For the production of indole-3-acetic acid (IAA), Zn-solubilizing bacteria were inoculated into LB broth with 1% L-tryptophan as an IAA precursor, and the culture was grown for 96 h at 120 rpm and 30 ± 2 °C. Cells were separated from the suspension by centrifugation at 10,000 rpm for 10 min. Then, Salkowski’s reagent was added to the supernatant at a ratio of 1:1 (v/v), and the mixture was stored in the dark for 25 min. To determine the amount of IAA, the absorbance at 530 nm was measured using a UV–vis double beam spectrophotometer (Thermo Scientific, GENESYS 10S UV–VIS, USA) and calibrated with a standard curve of pure IAA.

To measure the solubilization of inorganic phosphate, bacterial strain cultures were inoculated on Pikovskaya’s agar medium for 10 days at 30 ± 2 °C. Strains showing a halo zone around the colony were identified as P-solubilizing strains. For potassium solubilization, Aleksandrow agar medium was used. After incubation at 30 ± 2 °C for 7 days, the clear zone was observed. Chrome azurol S (CAS) medium was used for siderophore production according to a method described previously (Schwyn & Neilands, 1987). In brief, zinc-solubilizing bacteria were grown on NA agar at 30 ± 2 °C for 24 h and then point inoculated onto CAS agar. The cultures were incubated at 30 ± 2 °C for 5 days. A colony surrounded by light orange zones was considered to possess siderophore production ability. The production of ammonia (NH_3_) was evaluated by inoculating bacterial strains into 4% peptone broth and incubating them at 30 ± 2 °C for 96 h. One milliliter of Nessler’s reagent was added after incubation. A positive result for NH_3_-producing strains was the development of a yellow to dark brown hue.

### Evaluation of plant growth promotion effects

Zinc-solubilizing strains were examined for their ability to stimulate the growth of green soybean (*Glycine max* L. Merr. cv. Kamphaeng Saen MJ101) (Tropical Vegetable Research Center, Kasetsart University Kamphaeng Saen campus) in the greenhouse of Kasetsart University on the Kamphaeng Saen campus, Nakhon Pathom, Thailand. The experiment was performed from December 2021 to February 2022. For preparation of the bacteria, each isolate was cultivated in 150 mL of nutrient broth for 24 h at 30 ± 2 °C. Then, the cells were removed by centrifugation at 8,000 rpm for 10 min. The cell pellet was washed with sterilized deionized water and then adjusted to 10^8^ cell mL^−1^ with 0.85% normal saline.

The soybean seeds were surface-sterilized with 10% hypochlorite for 10 min and rinsed three times with sterile water (Kotabin et al., 2017). The seeds were then germinated in sterilized coco-peat for 10 days. The resulting seedlings were transplanted into 12-inch pots containing approximately 2 kg of soil and grown in a greenhouse. Sandy loam soil collected in Kamphaeng Saen district, Nakhon Pathom, Thailand, was used in the experiment. The properties of the soil were as follows: OM 16.98%, available phosphorus 1.21 g kg^−1^, exchangeable potassium 4.73 g kg^−1^, exchangeable zinc 12.53 mg kg^−1^, and pH 7.65. The greenhouse was illuminated with natural light (30 °C/25 °C day/night temperatures). Each treatment was performed using 15 pots. Before the flowering stage of soybean, 5 mL bacterial suspensions (10^8^ cell mL^−1^) were applied to each pot at 15 and 30 days after planting. Uninoculated seedlings were used as controls, and an application of 50 mg kg^−1^ zinc sulfate was used as a positive control. Plants were cultivated until they reached the fruiting period. After 70 days of growth, the plants were harvested, and the roots were washed carefully with running water. The lengths of the shoots and roots, the shoot and root dry weights, the numbers of pods and the numbers of grains were measured. Dry weight determinations were performed at 70 °C for 2 days.

### Statistical analysis

Statistical analysis was carried out by using Minitab 16 software (Minitab Inc.; Pennsylvania, USA). Data were analyzed using one-way analysis of variance followed by Tukey’s honestly significant difference test to verify significant differences between the treatments at *p* ≤ 0.05.

## Results

### Isolation of ZSB

Zn-solubilizing bacteria (ZSB) were isolated from the rhizosphere soils of peanuts, sweet potatoes and cassava growing in Chonburi and Kanchanaburi Provinces, Thailand (Table 1). The capacity of all isolates to solubilize zinc was evaluated using agar media supplemented with insoluble Zn compounds, *i.e.,* ZnO and ZnCO_3_. The occurrence of a halo zone indicates that the isolating organism can solubilize zinc. The halo zone diameter and zinc solubilization efficiency of the selected ZSB are presented in Table 2. Out of 121 isolates, five isolates with a zinc solubilization efficiency (ZSE) greater than 2.00 on ZnO supplemented medium (KAH109, KPIB103, KPIB105, KRME106 and KEX206) and the isolate KEX505 which appears to have strong zinc solubilization on ZnCO_3_ supplemented medium (ZSE 1.93) were selected for further study. All the selected strains can effectively solubilize insoluble Zn compounds, notably ZnO and ZnCO_3_, under the conditions of the assay. The isolates KAH109, KPIB103, KRME106 and KEX206 showed high ZSE in both ZnO- and ZnCO_3_-containing media. In comparison to that in ZnO-supplemented medium, the zone of solubilization in ZnCO_3_-supplemented medium was high. ZnO-containing medium, however, was associated with a higher ZSE than ZnCO_3_-supplemented medium. The isolate KAH109 exhibited the highest ZSE in ZnO-supplemented medium (2.84 ± 0.47). Therefore, 0.1% ZnO was used for the Zn solubilization study in liquid medium. The bacterial isolates solubilized insoluble ZnO at concentrations ranging from 36.12 to 62.89 mg L^−1^ soluble Zn (Table 3). Isolate KAH109 showed the maximum solubilization of Zn (62.79 mg L^−1^). The bacterial isolates altered the pH of the culture medium to between 5.36 and 6.40. The decrease in the pH of the culture medium was proportional to the quantity of soluble Zn produced by ZSB.

**Table 1:** Bacteria isolated from rhizosphere soil from diverse plant species.

Plant	Location	Number of isolate	Isolate code
Cassava	N 12° 44′12.21″E 101° 18′51.67″	9	KAH101, KAH102, KAH103, KAH104, KAH105, KAH106, KAH107, KAH108, KAH109
Sweet potato	N 12° 44′11.58″E 101° 18′53.27″	8	KOIB101, KOIB102, KOIB103, KOIB104, KOIB105, KOIB106, KOIB107, KOIB108
Sweet potato	N 12° 44′12.20″E 101° 18′52.64″	3	KOIB201, KOIB202, KOIB203
Peanut	N 12° 44′12.59″E 101° 18′52.32″	12	KPIB101, KPIB102, KPIB103, KPIB104, KPIB105, KPIB106, KPIB107, KPIB108, KPIB109, KPIB110, KPIB111, KPIB112
Peanut	N 12° 44′12.09″E 101° 18′53.25″	4	KPIB201, KPIB202, KPIB203, KPIB204
Peanut	N 12° 44′12.31″E 101° 18′51.78″	4	KPIB301, KPIB302, KPIB303, KPIB304
Peanut	N 12° 44′42.13″E 101° 20′52.42″	7	KRME101, KRME102, KRME,103, KRME104, KRME105, KRME106, KRME107
Cassava	N 14° 11′54.3″E 99° 35′44.7″	4	KEX101, KEX102, KEX103, KEX104
Cassava	N 14° 11′47.2″E 99° 35′47.0″	9	KEX201, KEX202, KEX203, KEX204, KEX205, KEX206, KEX207, KEX208, KEX209
Cassava	N 14° 11′49.9″E 99° 35′46.1″	13	KEX301, KEX302, KEX303, KEX304, KEX305, KEX306, KEX307, KEX308, KEX309, KEX310, KEX311, KEX312, KEX313
Cassava	N 14° 11′53.5″E 99° 35′01.7″	4	KEX401, KEX402, KEX403, KEX404
Cassava	N 14° 11′52.2″E 99° 35′02.6″	13	KEX501, KEX502, KEX503, KEX504, KEX505, KEX506, KEX507, KEX508, KEX509, KEX510, KEX511, KEX512, KEX513
Cassava	N 14° 11′31.9″E 99° 34′31.1″	3	KEX601, KEX602, KEX603,
Cassava	N 14° 01′30.9″ E 99° 58′43.6″	5	KSM101, KSM102, KSM103, KSM104, KSM105
Cassava	N 14° 01′31.7″E 99° 58′44.3″	10	KSM201, KSM202, KSM203, KSM204, KSM205, KSM206, KSM207, KSM208, KSM209, KSM210
Cassava	N 14° 01′30.5″E 99° 58′43.0″	4	KSM301, KSM302, KSM303, KSM304
Cassava	N 14° 01′21.9″E 99° 59′01.1″	5	KPEP101, KPEP102, KPEP103, KPEP104, KPEP105
Cassava	N 14° 01′22.2″E 99° 59′00.7″	4	KPEP201, KPEP202, KPEP203, KPEP204

**Table 2:** Zinc solubilizing efficiency of different ZSB isolates in solid medium using insoluble zinc compounds.

Isolates	Zn solubilization					
	ZnO			ZnCO_3_		
	Colony diameter (cm)	Zone of clearance (cm)	S.E.	Colony diameter (cm)	Zone of clearance (cm)	S.E.
KAH109	0.37 ± 0.06	1.05 ± 0.26	2.84 ± 0.47	0.79 ± 0.18	1.80 ± 0.33	2.27 ± 0.10
KPIB103	0.54 ± 0.15	1.17 ± 0.17	2.16 ± 0.17	0.54 ± 0.03	1.17 ± 0.06	2.16 ± 0.02
KPIB105	0.38 ± 0.07	0.90 ± 0.36	2.33 ± 0.45	0.68 ± 0.08	1.21 ± 0.25	1.76 ± 0.17
KRME106	0.49 ± 0.07	1.09 ± 0.35	2.20 ± 0.37	0.83 ± 0.15	1.66 ± 0.31	2.00 ± 0.08
KEX206	0.53 ± 0.03	1.07 ± 0.14	2.01 ± 0.26	0.52 ± 0.15	1.24 ± 0.01	2.39 ± 0.13
KEX505	0.33 ± 0.02	0.43 ± 0.08	1.32 ± 0.14	0.81 ± 0.22	1.53 ± 0.21	1.93 ± 0.25

**Table 3:** Zinc solubilizing efficiency of different ZSB isolates in liquid medium using ZnO. Data are statistically analyzed using one-way ANOVA followed by Tukey’s honestly test. Mean ± SD values with different lowercase superscripts are significantly (*p* < 0.05) different.

Isolate	Soluble zinc (mg L^−1^)	pH of culture broth
KAH109	62.89 ± 1.12^a^	5.36 ± 0.03
KPIB103	46.51 ± 1.23^b^	5.79 ± 0.22
KPIB105	50.34 ± 3.21^b^	5.59 ± 0.10
KRME106	44.47 ± 4.01^bc^	6.21 ± 0.02
KEX206	41.96 ± 2.45^bc^	6.22 ± 0.04
KEX505	36.71 ± 0.77^c^	6.40 ± 0.06
Control	12.25 ± 0.23^d^	6.85 ± 0.02

### Characterization and identification

**Figure 1:**
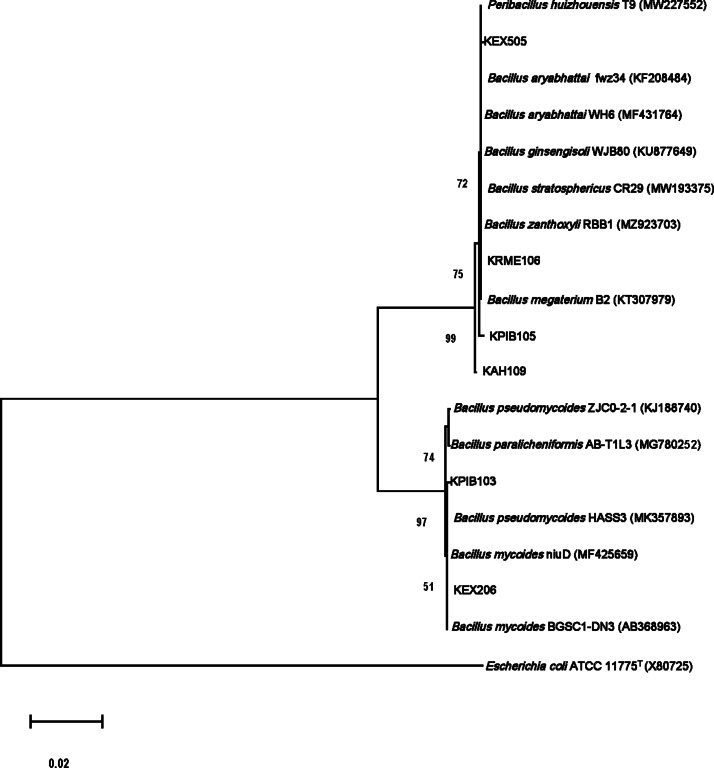
Molecular phylogenetic tree analysis based on 16S rRNA gene sequences of KAH109, KPIB103, KPIB105, KRME106, KEX206 and KEX505 relative to type strains of other *Bacillus* species. Maximum likelihood method was employed to construct a tree. *E. coli* ATCC1175^T^.

The isolates were gram-positive, rod-shaped, and endospore-producing bacteria. Based on 16S rRNA gene sequencing, the ZSB strains belonged to the phylum Firmicutes (Table 3). The isolate KAH109 showed 100% shared sequence homology with *Priestia megaterium*. The isolate KPIB103 showed 99.79% shared sequence homology with *Bacillus mycoides*. KPIB105 showed maximum shared similarity (99.79%) with *Priestia megaterium*. KRME106 exhibited maximum shared similarity (99.86%) with *Priestia aryabhattai*. The isolate KEX206 showed maximum similarity (99.79%) with *Bacillus mycoides*. KEX505 displayed maximum shared similarity (99.93%) with *Priestia aryabhattai*. The 16S rRNA gene sequences of the isolates were submitted to NCBI GenBank under accession numbers OL721893, OM011975, OL721889, OL721892, OL721880 and OL721885. On the basis of 16S rRNA gene sequences, a phylogenetic tree depicting the relationships between isolates and related bacteria was created (Fig. 1).

### Plant growth-promoting traits

As shown in Table 4, the ZSB were evaluated for different plant growth promotion characteristics, including IAA production, siderophore production, NH_3_ production, and phosphorus and potassium solubilization. With the exception of isolate KPIB103, all isolates produced IAA over a range of 11.36 to 33.44 mg L^−1^. The isolate KEX505 exhibited potassium solubilization. All the isolates exhibited NH_3_ production. The isolates KPIB103 and KRME106 could solubilize inorganic phosphate. None of the isolates were able to produce siderophores. Although the isolates KAH109 and KPIB105 were identified as *P. megaterium*, the zinc solubilization efficiency and IAA production level of the isolate KAH109 were obviously greater than those of the isolate KPIB105.

### Plant growth promotion in pot experiments

According to the above results on zinc solubilization efficiency, IAA production and potassium solubilization (Table 4), *Priestia megaterium* KAH109 and *Priestia aryabhattai* KEX505 were selected to examine their capabilities of promoting plant growth in green soybean. Inoculation with *Priestia megaterium* KAH109 promoted the growth of green soybeans by significantly increasing the plant dry weight. *P. megaterium* KAH109 and *P. aryabhattai* KEX505 inoculation increased root length by 19.12% and 20.61%, respectively, as compared to the uninoculated control (Table 5). The plant dry weights of green soybeans inoculated with *P. megaterium* KAH109 and *P. aryabhattai* KEX505 significantly increased by 26.96% and 8.79%, respectively. Regarding production yield, inoculating *P. megaterium* KAH109 and *P. aryabhattai* KEX505 significantly increased the number of grains per plant by 48.97% and 35.29%, respectively.

**Table 4:** Plant growth promoting attributes zinc solubilizing bacteria.

Isolate	IAA production (mgL^−1^)	Phosphate solubilization (SE)	Potassium solubilization (SE)	Siderophore production	NH_3_ production	Result of identification		
						Species	% Similarity	Accession no.
KAH109	33.44 ± 0.55	–	–	–	+	*Priestia megaterium*	100.00	OL721893
KPIB103	–	1.09 ± 0.00	–	–	+	*Bacillus mycoides*	99.79	OM011975
KPIB105	20.58 ± 3.19	–	–	–	+	*Priestia megaterium*	99.79	OL721889
KRME106	31.62 ± 1.66	1.10 ± 0.05	–	–	+	*Priestia aryabhattai*	99.86	OL721892
KEX206	11.36 ± 2.44	–	–	–	+	*Bacillus mycoides*	99.79	OL721880
KEX505	17.24 ± 3.33	–	1.10 ± 0.03	–	+	*Priestia aryabhattai*	99.93	OL721885

**Notes.**

Note: + positive, − negative

**Table 5:** Effect of ZSB on growth and yield of green soybean (*Glycine max* L. Merr) in greenhouse experiment. Data are statistically analyzed using one-way ANOVA followed by Tukey’s honestly test. Mean ± SD values with different lowercase superscripts are significantly (*p* < 0.05) different Sandy loam soil was used in the experiment. Green soybeans were cultivated during December 2021 to February 2022.

	Shoot length (cm)	Root length (cm)	Plant DW (g/plant)	Number of pod (Pod/plant)	Number of grain (Grain/plant)
Control	26.67 ± 2.87^a^	42.933 ± 4.42^b^	5.394 ± 0.843^a^	7.33 ± 1.29^c^	21.93 ± 4.51^b^
Zinc sulfate (50 mg kg^−1^)	28.33 ± 4.03^a^	50.35 ± 5.07^a^	5.754 ± 0.419^a^	10.40 ± 0.99^b^	31.73 ± 6.58^a^
*Priestia megaterium* KAH109	26.87 ± 2.87^a^	51.14 ± 5.94^a^	6.848 ± 0.871^b^	11.47 ± 1.46^b^	32.67 ± 6.44^a^
*Priestia aryabhattai* KEX505	28.82 ± 4.38^a^	51.78 ± 5.37^a^	5.868 ± 0.931^a^	16.00 ± 3.51^a^	29.67 ± 5.26^a^

## Discussion

In the present study, bacteria were isolated from the rhizosphere soil of peanuts, sweet potatoes and cassava and screened on Bunt and Rovira’s agar medium supplemented with ZnO and ZnCO_3_ to investigate their ability to solubilize zinc. The results revealed that out of 121 isolates, 6 isolates showed strong solubilization of ZnO and ZnCO_3_. The zinc solubilization efficiency of the bacterial isolates on agar media containing ZnO ranged from 1.32 to 2.84 (Table 2). The maximum solubilization of zinc was obtained with *Priestia megaterium* KAH109. In previous studies, zinc solubilization efficiency varied among bacterial strains (Bhatt & Maheshwari, 2020; Kushwaha et al., 2021). In this work, the zinc solubilization efficiency of ZnO appeared to be comparable to that of ZnCO_3_. In contrast to earlier studies, which found the highest zinc solubilization efficiency for zinc oxide, this study demonstrates that zinc sulfate is associated with the highest solubilization efficiency (Ramesh et al., 2014; Mumtaz et al., 2017). Saravanan, Subramoniam & Raj (2003) reported that *Bacillus* sp. isolated from zinc ore sphalerite can solubilize ZnO, ZnCO_3_ and ZnS. Therefore, differences in zinc solubilization efficacy among zinc compounds might be attributed to variations in the environments in which they were isolated. *Bacillus* is one of the most explored genera of bacteria due to its ubiquity in nature and ability to solubilize zinc (Sharma et al., 2012; Ramesh et al., 2014). Our results are consistent with previous findings that a significant reduction in the pH of culture medium inversely correlates with the amount of soluble zinc released. Zinc solubilization by the isolates may be due to the formation of organic acids. There are several strategies for dissolving zinc, including the excretion of metabolites such as organic acids, proton expulsion, and the production of chelating agents. Several microbes generate organic acids in the soil, which bind zinc cations and lower the pH of the surrounding soil. Furthermore, the anions can chelate zinc and improve zinc availability. It has been reported that lactic, acetic, succinic, formic, isobutyric, and isovaleric acid generation by microbial isolates is a key solubilization process (Mumtaz et al., 2019). Simine, Sayer & Gadd (1998) found that gluconic acid and 2-ketogluconic acids produced in the culture broth aided in the solubilization of zinc phosphate by *Pseudomonas fluorescens*. A decrease in the pH and acidification of the medium were noted in all cases. Metal cations can be complexed by the carboxylic groups of organic acids, and anions can be displaced to make ZnO more soluble. In addition, the formation of inorganic acids such as sulfuric acid, nitric acid, and carbonic acid might aid in solubilization. However, in certain potent strains, the pH did not drop significantly, indicating that additional processes may be functioning in those strains (Desai et al., 2015). In addition to zinc solubilization, these zinc-solubilizing bacterial isolates exhibit other plant growth-promoting traits, such as indole-3-acetic acid (IAA) and NH_3_ production and potassium solubilization (Table 4). Therefore, these ZSB isolates could support plant growth. Based on 16S rDNA sequence analysis, four of the potent isolates belonged to the genus *Priestia*, and the other two isolates belonged to the genus *Bacillus* (Table 4). A gram-positive bacterium identified as a *Priestia* species (formerly known as *Bacillus*) is abundant in soil. It has the ability to produce resistant endospores, which allows it to survive in stressful environments. *Priestia* species have been widely reported to enhance plant growth through several mechanisms (Kordatzaki et al., 2022).

Zinc is not only an essential nutrient for cofactors of important enzymes, such as superoxide dismutase and carbonic anhydrase, but also plays an important role in IAA and tryptophan biosynthesis. Zn deficiency results in the rapid degradation of IAA, a decrease in carbon dioxide fixation and the leakage of small organic molecules such as potassium ions, amino acids and sugars (Cakmak, Marschner & Bangerth, 1989). Zinc and potassium ions control the biosynthesis of carbohydrates and their transport to the apical meristem and storage tuber. For soybean cultivation, zinc and potassium fertilizers are essentially used during the flowering stage (Joy et al., 2015; Popp, Slaton & Roberts, 2020). ZSB can mobilize unavailable zinc, resulting in increased zinc and potassium assimilation, plant growth and plant yield. In this study, *P. megaterium* KAH109, which exhibited the highest zinc solubilization activity and IAA and NH_3_ production, and *P. aryabhattai* KEX505, which showed zinc solubilization activity, IAA and NH_3_ production and potassium solubilization activity, were selected and studied for their effects on green soybean growth and grain yield. The inoculation of both strains into the soil of green soybeans during the greenhouse experiment had a positive impact on plant growth and productivity compared with that in the uninoculated and positive controls, in which 50 mg kg^−1^ zinc sulfate was applied. Inoculation with *P. megaterium* KAH109 and *P. aryabhattai* KEX505 did not significantly increase shoot and root lengths, which contrasts with other findings by Ramesh et al. (2014). However, *P. megaterium* KAH109 significantly increased plant dry weight by 26.96%, as well as the number of grains per plant by 48.97% (Table 5). Both strains apparently increased lateral root growth, which is beneficial for plants because it increases soil water and nutrient uptake, reducing the need for fertilizer use in the field (Lynch & Brown, 2012). Colonization of plant roots is advantageous for both the bacterium and the host plant. In *B. subtilis*, chemotaxis plays an important role in locating and colonizing young roots (Allard et al., 2016). Chemoreceptors found in *B. subtilis* allow it to locate a specific habitat: plant roots (Glekas et al., 2012; Yang et al., 2015). Some *Bacillus* species release ammonia from nitrogenous organic matter (Hayat et al., 2010). According to the laboratory results, the pH of the culture medium decreases during zinc solubilization, implying that the zinc in the rhizosphere soil is reallocated across native zinc pools, resulting in increased availability of zinc and other micronutrient cations for plants. Khande et al. (2017) evaluated prospective zinc-solubilizing bacteria for improved nutrition and zinc uptake in *Zea mays* L., as well as zinc-solubilizing *Bacillus* strains that affect the growth, yield, and zinc bioaugmentation of soybean and wheat. The production of auxins (IAA) and potassium solubilization activity are well known for their ability to improve plant growth (Poveda & González-Andrés, 2021). Although rhizosphere soil was the habitat of ZSB in this study, seed coating and foliar spraying can be used as additional planting methods. Furthermore, applying ZSB in consortiums or in combination with chemical fertilizer will increase zinc availability and benefit for plant growth and production.

## Conclusions

In conclusion, we isolated and screened the potential of Zn-solubilizing bacteria (ZSB) in the rhizosphere soils of sweet potato, peanuts and cassava. The selected strains *Priestia megaterium* KAH109 and *P. aryabhattai* KEX505 showed a high zinc solubilization ability and exhibited various plant growth promotion activities. The inoculation of *P. megaterium* KAH109 or *P. aryabhattai* KEX505 into the soil of green soybean plants significantly increased biomass and grain yield compared to those of the uninoculated control. Our findings suggested that both *Priestia* strains might serve as bioinoculants to enhance plant development and yield. In future studies, the effect of co-inoculation or seed priming with these strains will be explored. In addition, correlations will be examined between the populations of ZSB, the amount of soil available zinc, and the activities in the rhizosphere soil that promote plant growth both in greenhouse and field trials.

##  Supplemental Information

10.7717/peerj.15128/supp-1 Supplemental Information 1 ZSE on agar media

10.7717/peerj.15128/supp-2 Supplemental Information 2 ZSE in liquid medium

10.7717/peerj.15128/supp-3 Supplemental Information 3 IAA PSE and KSE

10.7717/peerj.15128/supp-4 Supplemental Information 4 Soybean growth

10.7717/peerj.15128/supp-5 Supplemental Information 5 Gene Sequences

## Additional Information and Declarations

### DNA Deposition

The following information was supplied regarding the deposition of DNA sequences: The gene sequences are available at NCBI GenBank: OL721893, OM011975, OL721889, OL721892, OL721880 and OL721885.

### Data Availability

The following information was supplied regarding data availability: The raw data is available in the Supplemental Files.
